# Ageing Effects in Mounting Media of Microscope Slide Samples from Natural History Collections: A Case Study with Canada Balsam and Permount^TM^

**DOI:** 10.3390/polym13132112

**Published:** 2021-06-27

**Authors:** Thomas Schmid, Julia Hidde, Sophie Grünier, Robert Jungnickel, Petra Dariz, Jens Riedel, Birger Neuhaus

**Affiliations:** 1 Bundesanstalt für Materialforschung und -Prüfung, 12489 Berlin, Germany; ailuj25@web.de (J.H.); sophie.gruenier@gmail.com (S.G.); robert.jungnickel@posteo.de (R.J.); Jens.Riedel@bam.de (J.R.); 2 School of Analytical Sciences Adlershof (SALSA), Humboldt-Universität zu Berlin, 10115 Berlin, Germany; petra.dariz@bfh.ch; 3 Bern Universiy of Applied Sciences, Bern University of the Arts, Institute Materiality in Art and Culture, 3027 Bern, Switzerland; 4 Museum für Naturkunde, Leibniz Institute for Evolution and Biodiversity Science, 10115 Berlin, Germany; Birger.Neuhaus@mfn.berlin

**Keywords:** ageing, conservation, deterioration, microscope slides, mounting media, natural history museum, Raman spectroscopy, UV–Vis spectroscopy, accelerated ageing

## Abstract

Microscope slide collections represent extremely valuable depositories of research material in a natural history, forensic, veterinary, and medical context. Unfortunately, most mounting media of these slides deteriorate over time, with the reason for this not yet understood at all. In this study, Raman spectroscopy, ultraviolet–visible (UV–Vis) spectroscopy, and different types of light microscopy were used to investigate the ageing behaviour of naturally aged slides from museum collections and the experimentally aged media of Canada balsam and Permount™, representing a natural and a synthetic resin, respectively, with both being based on mixtures of various terpenes. Whereas Canada balsam clearly revealed chemical ageing processes, visible as increasing colouration, Permount™ showed physical deterioration recognisable by the increasing number of cracks, which even often impacted a mounted specimen. Noticeable changes to the chemical and physical properties of these mounting media take decades in the case of Canada balsam but just a few years in the case of Permount™. Our results question whether or not Canada balsam should really be regarded as a mounting medium that lasts for centuries, if its increasing degree of polymerisation can lead to a mount which is no longer restorable.

## 1. Introduction

Worldwide, natural history collections, forensic institutes, veterinary institutes, universities, and medical hospitals house millions of microscope slides with entire specimens, body parts of a specimen, or histological sections for both reference and research purposes [1,2,3]. Such reference collections are indispensable for the description of new species by comparing them with known species, identifying unknown specimens, for teaching, and for studying diseases and parasites. Type material must nowadays be stored as part of the process of the description of a new species in a biological reference collection, usually in a natural history museum [4]. This type material represents the new species and must be kept for centuries to follow. Therefore, it is scientifically extremely valuable and should be studied every time a closely related new species is described.

It has long been known that many mounting media do not last longer than a few years, sometimes decades. A first macroscopic inspection of the microscope slides in the collection “Vermes” at the Museum für Naturkunde Berlin revealed, in particular, cavities and crystallisation in different mounting media [3]. Di Giacomo [2] observed similar phenomena in 3% to far more than 65% of the slides during a survey of different collections at the National Museum of Natural History, Smithsonian Institution. The severity of problems seemed to depend on the mounting media most frequently used. In particular, glycerol gelatin, polyvinyl lactophenol, Permount^TM^, and Hoyer’s medium were among the least recommendable mounting media [2,3]. Unfortunately, information about which mounting medium was applied to a specific slide is not always available from the label, a protocol, or a catalogue, so salvaging slides often turns out to be a task of time-consuming trial and error [2,3,5,6].

Deteriorating mounting media comprise inter alia Aquatex^®^, Fluoromount G^TM^, polyvinyl lactophenol, gum-chloral based media like Berlese’s, Faure’s, and Hoyer’s mounting media, and Permount^TM^ [1,3,7,8,9,10,11,12,13,14,15,16,17,18]. The deteriorative processes may lead to yellowing in natural resins and to rainbow-coloured discolouration, cracking, crystallisation, shrinkage on drying or possibly on loss of a plasticiser, detachment of the coverslip, segregation of the ingredients in synthetic polymers, as well as ongoing maceration of a specimen to a degree that the specimen cannot be traced with a light microscope anymore (see summary in Ref. [3]). Few media seem to last at least 150 years like Canada balsam, more than 50 years such as Euparal, or are totally reversible and do not seem to deteriorate like glycerol-paraffin mounts [1,3] (but for problems with the evaporation of glycerol in glycerol-paraffin mounts, see Refs. [19,20]). Although Canada balsam yellows and darkens over time, this is in biological samples usually overcome by illuminating the slide with a higher intensity of transmitted light [1]. Permount^TM^ has been found to be one of the most problematic mounting media, with rapidly developing concentric rings of cracks and yellowing [7,8,12,18,21,22,23]. Deterioration of this medium has been related to “diffusion of solvent outward, and perhaps also of atmospheric oxygen inward from the coverslip margin”, to fluctuating humidity and temperatures [7] (p. 142), and to the lack of an oil-soluble antioxidant like 2-6-di-*tert*-butyl-*p*-cresol [8] (p. 419). Based on our own experience, even briefly applied mechanical stress to the microscope mount seems to trigger cracking and subsequent yellowing within about two weeks [23].

The results of the deterioration processes of mounting media on microscope slides are easily observable at an advanced stage with the naked eye (cracking, shrinking, yellowing) or a microscope (crystallisation, cavities, segregated parts of the media). Modern methods have very rarely been applied to at least identify an unknown mounting medium [2,5,6,22,24]. Garner and Horie [5] (p. 93) identified a mounting medium and coverslip seal with the help of X-ray fluorescence and infrared spectroscopy, but mentioned these methods only briefly in a footnote. Destructive methods like Fourier-transform infrared spectroscopy (FTIR) and attenuated total reflection Fourier-transform infrared spectroscopy (ATR–FTIR) were applied to identify unknown mounting media [2,6]. A proof-of-concept study was done by Schmid et al. [24] to demonstrate that mounting media can be identified by non-destructive Raman microscopy. Whereas this latter method allows operating through the glass of the coverslip, FTIR and ATR–FTIR are destructive methods in the sense that the coverslip has to be removed, because infrared radiation cannot penetrate glass. However, a systematic approach to document chemical changes or to understand ageing phenomena in mounting media, as suggested by Neuhaus et al. [3], is lacking entirely.

In this paper we report on ageing effects in both a long-lasting mounting medium, Canada balsam, and a quickly deteriorating medium, Permount^TM^. While both media are based on mixtures of various terpenes, Canada balsam is a natural resin and Permount^TM^ a synthetic polymer [3]. We studied naturally aged microscope slides from different collections and museums with the help of Raman spectroscopy, ultraviolet–visible (UV–Vis) spectroscopy, and different types of light microscopy to identify the mounting medium, measure the absorbance, and relate the deterioration artefacts to physicochemical changes during ageing. Furthermore, we present the results of an accelerated ageing study of test slides of Canada balsam and Permount^TM^.

## 2. Materials and Methods

### 2.1. Historical Microscope Slide Samples

The 12 studied historical microscope slide samples showing different degrees and types of damages are listed in Table 1.

### 2.2. Mounting Media and Sample Preparation for Accelerated Ageing Experiments

Three batches of Canada balsam were provided by the Museum für Naturkunde Berlin that were purchased from Merck (termed ‘supplier 1’ below) in the middle of the 1990s and in the early 2010s, respectively, as well as from Carl Roth (‘supplier 2’) in the early 2010s. The accelerated ageing studies were performed with the latter. Permount^TM^ was purchased from Fisher Scientific (Fisher Scientific GmbH, Schwerte, Germany) directly at the beginning of the study. For each ageing experiment, a few droplets of the medium were transferred onto a glass slide by using a spatula without adding a cover slip. Each sample was cured in the dark at normal lab atmosphere for 5 days prior to the ageing experiments described below.

### 2.3. Accelerated Ageing Experiments

For accelerated ageing under UV irradiation, a Lightningcure LC8 spot light (-01A type, Hamamatsu Photonics, Hamamatsu, Japan) UV source based on a mercury–xenon lamp having an emission spectrum centred at λ = 365 nm and a power density of 4500 mW/cm^2^ (at 365 nm) was employed. For the accelerated ageing experiments described here, the power was attenuated to 30%.

Accelerated ageing under oxidative conditions was performed by placing glass slide samples onto the ceramic plate of a glass desiccator (without drying agent), which was heated in a conventional lab drying oven (WST 5010, VEB MLW, Leipzig, German Democratic Republic). The temperature was controlled with a thermometer (±1 K precision) placed next to the desiccator.

### 2.4. Spectroscopic Measurements and Microscopic Imaging

UV–Vis spectra were acquired using a V-650 UV–Vis spectrophotometer (Jasco Deutschland GmbH, Pfungstadt, Germany). Transmittance through the empty sample chamber was employed as the reference. Spectra in the wavelength range of 300 nm to 700 nm were acquired with a scanning speed of 400 nm per minute.

Raman spectra were acquired using a LabRam HR 800 Raman spectrometer (Horiba Jobin-Yvon, Bensheim, Germany) coupled with a BX41 upright microscope (Olympus, Hamburg, Germany). The system enables observation of the sample under normal white-light illumination in order to choose appropriate measurement spots. During Raman measurements, a HeNe laser with a wavelength of 632.8 nm and a power at the sample of 9 mW was used for excitation. The laser was focused by a 50×/N.A. = 0.55 long-working distance objective (with N.A. denoting the numerical aperture), and the scattered light was collected through the same lens. In the spectrometer, the collected light was dispersed using a 300 mm^−1^ grating and projected onto a 1024 × 256 pixel CCD detector (Symphony, Horiba Jobin-Yvon, N_2_-cooled, operated at −126 °C). Typical measurement time was 60 s per spectrum, and in case of saturation of the detector (more than 65,535 counts), was split into several accumulations, e.g., 2 × 30 s or 4 × 15 s.

Micrographs shown in Figure 3, Figure 4 and Figure 15 in Section 3 below were acquired by using the BX41 microscope (Olympus) of the Raman microscopy system. All other reflected (brightfield or darkfield) or transmitted light micrographs were acquired by employing a AxioScope.A1 MAT microscope (Carl Zeiss Microscopy GmbH, Jena, Germany).

## 3. Results and Discussion

### 3.1. Identification of the Mounting Media of Historical Microscope Slide Samples

**Figure 1:**
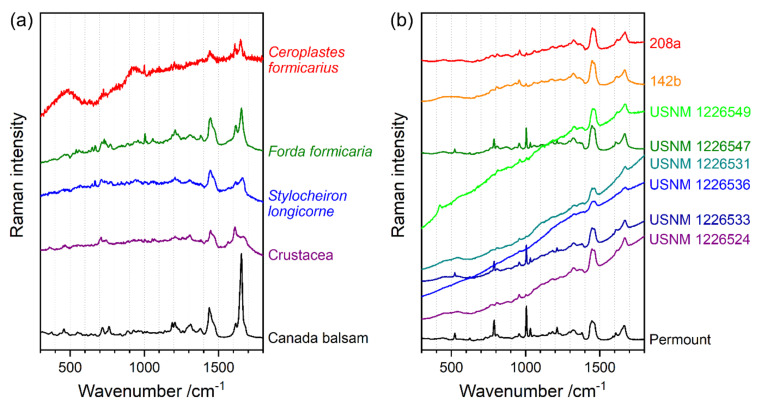
Identification of mounting media: (**a**) Canada balsam, and (**b**) Permount™; USNM followed by catalogue number of the United States National Museum, Washington, D.C., Smithsonian Institution, *Cateria gerlachi* (Kinorhyncha); *Sorex araneus* (Mammalia), # 142b and *Ctenodactylus gundi* (Mammalia), # 208a, numbers refer to the Hubrecht Collection. The black traces at the bottom represent Raman reference spectra of pure mounting media.

The mounting media of 12 microscope slide samples from natural history collections were determined using a Raman spectroscopy method previously described by Schmid et al. [24]. Raman spectra contain bands (i.e., peaks) corresponding to the Raman-active vibrations of molecules, with wavenumbers (unit cm^−1^) corresponding to their vibrational frequencies. Thus, Raman spectra are very specific spectroscopic fingerprints of molecular structures, and molecules can be identified by the comparison of wavenumber positions and the shapes of peaks with reference spectra. Therefore, an important part of the previous study [24] was to build up a spectral library by measuring the Raman spectra of 17 pure mounting media from the laboratory of the Museum für Naturkunde Berlin. The Raman spectra of the 12 microscope slide samples investigated in the present study were compared with the spectral library, and 4 were assigned to Canada balsam (Figure 1a), while the other 8 were obviously mounted in the synthetic medium Permount^TM^. As described previously [24], unspecific autofluorescence emission from the aged media interferes with the Raman spectra, in all cases resulting in a raised baseline, which is a part of a spectrally broad fluorescence band. Spectra in Figure 1 were scaled and offset for better comparability.

### 3.2. Typical Damages in Historical Microscope Slide Samples

#### 3.2.1. Yellowing

**Figure 2:**
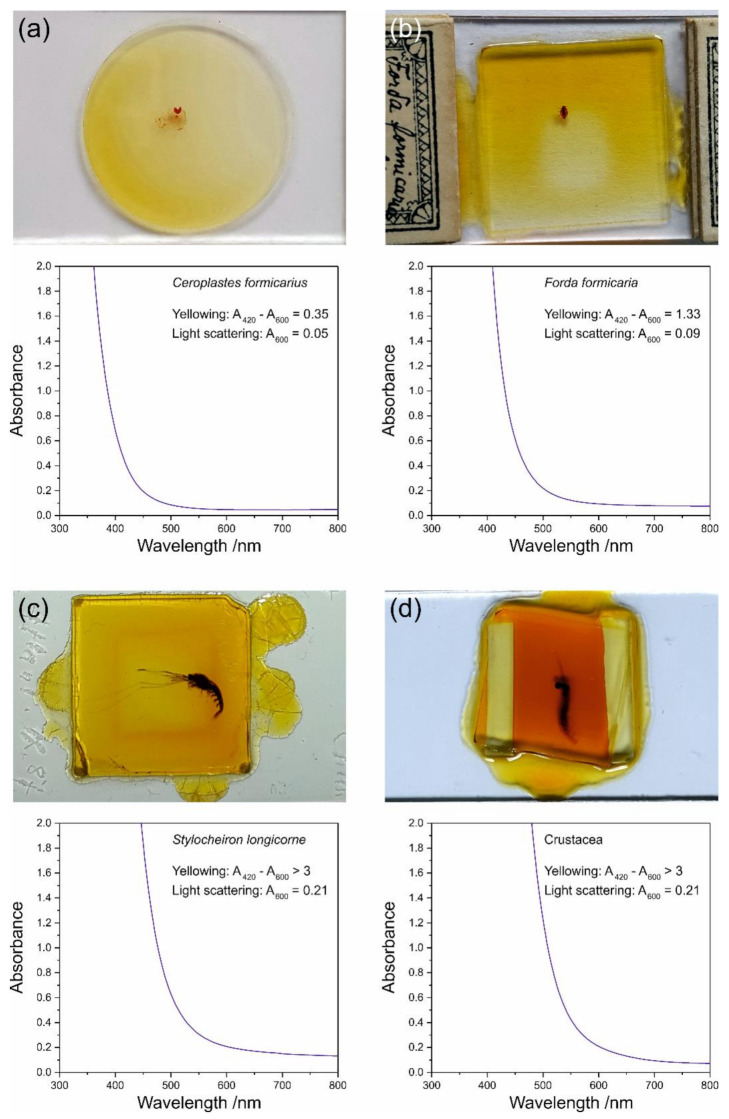
Yellowing as an ageing effect of Canada balsam observed in samples from natural history collections: (**a**) *Ceroplastes formicarius*, (**b**) *Forda formicaria*, (**c**) *Stylocheiron longicorne*, and (**d**) Crustacea.

Four samples under study from the collection of the Museum für Naturkunde Berlin showed strong yellowing of the medium: two Insecta (*Ceroplastes formicarius* and *Forda formicaria*) and two Crustacea samples (*Stylocheiron longicorne* and a sample of an undetermined species, simply termed ‘Crustacea’ in this study). The mounting medium of all four samples is Canada balsam (see identification by Raman spectroscopy in Figure 1a). Figure 2 shows photographs and UV–Vis spectra of these samples.

The absorbance measurements were performed by detecting and spectrally analysing a beam of white light transmitted by approximately the centre of each sample (if not affected by the animal). The absorbance is defined as lg (*I*_0_/*I*), with *I* denoting the intensity of transmitted light, while *I*_0_ refers to the beam of white light not affected by the sample, which was determined by measuring the transmittance of the empty measurement chamber. All spectra show strong absorbance in the ultraviolet <400 nm and a quasi-exponential decay towards the visible light range. Yellowing is caused by a shift of the falling edge of the absorbance spectrum into the visible range, with absorbance in the violet range resulting in yellow colour and absorbance in the blue range yielding orange colour in the medium. For quantitative evaluation of the UV-Vis spectra, we defined yellowing and light scattering factors in accordance with the work of de la Rie [26]:

Yellowing = *A*_420_ − *A*_600_, (1)

Light Scattering = *A*_600_, (2)

with *A*_420_ and *A*_600_ denoting the absorbances at 420 nm and 600 nm, respectively. This definition is based on the observations that yellowing leads to a shift of the absorbance edge into the violet range equalling an increase of the violet absorbance at 420 nm, while light scattering adds an offset to the whole spectrum, leading to a shift of the baseline parallel to the abscissa and thus, an increase of the absorbance, for example, in the orange spectral range around 600 nm. In contrast to de la Rie’s yellowness factor, we used the absorbance at 600 nm instead of 580 nm for correcting the influence of light scattering, and consistently used the absorbance at that wavelength as the light scattering factor. This change was applied because we observed an influence of yellowing onto the 580-nm absorbance in samples that were subject to strong colour changes towards red.

In all four samples we observed significant yellowing, ranging from 0.35 (*Ceroplastes formicarius*) up to >3 (*Stylocheiron longicorne* and Crustacea). As an absorbance of 3 equals a transmittance of 0.1%, which is close to the detection limit of a typical UV–Vis spectrometer, samples with yellowing >3 cannot be quantitatively analysed by this method. In the same samples, no influence of light scattering was observed yielding values ranging from 0.05 to 0.21. Even the highest light scattering factors observed in the samples *Stylocheiron longicorne* and Crustacea were most likely not due to light scattering, but rather due to a slight influence of the strong colouring onto the absorbance at 600 nm. This was confirmed by microscopic observations of these samples, which did not reveal any light scattering structures, such as crystals or cracks (data not shown).

The photographs in Figure 2a–c reveal an inhomogeneous distribution of the colouring, which correlates with the shape of the cover slip, i.e., a circular distribution of dark yellow medium along the edge of the cover glass in the sample *Ceroplastes formicarius*, and rectangular distributions of dark colours in the samples *Forda formicaria* and *Stylocheiron longicorne*, with the brightest medium in the central parts of all samples. This might be explained by the diffusion of oxygen from the edges into the media, which is potentially involved in chemical reactions leading to the observed yellowing effects. The ageing of fossil resins can be explained by several chemical transformation processes, including polymerisation, which is at least partly accompanied by a loss of isolated (i.e., olefinic or non-aromatic) double bonds [27], as well as aromatisation of penta- and hexacyclic hydrocarbon rings in terpenoids [28]. The latter process might explain the observed yellowing effect during the relatively short-term ageing of Canada balsam when compared to fossil resins, because optical absorption in the visible range by organic molecules is often related to chromophores with extended linear conjugated and/or aromatic π systems. Conversion of an aliphatic –CH_2_– into a =CH– group as part of a conjugated system raises the oxidation state of carbon from −2 to −1. Thus, aliphatic or olefinic ring aromatisation is an oxidation reaction in which atmospheric oxygen can act as an oxidant; further, due to dehydrogenisation of the ring, oxygen can be converted into water, and has been shown to be directly incorporated into functional groups of terpenoid resins [29,30]. Such chemical processes, and especially polymerisation, raise the question of for how long a mounting medium may be dissolvable again by organic solvents in case the mount has to be restored because of increasing colouration and difficulties in studying a mounted animal.

#### 3.2.2. Crack Formation

Cracks and crystals in mounting media, only observable by microscopic observation, are often difficult to distinguish, as radial cracks starting from one point or crackly areas showing light diffraction effects (see reflected light micrographs in Figure 5 below) can be easily misinterpreted as crystals, as has been done by Neuhaus and Kegel [18]. Here, we define cracks as purely physical changes within a mounting medium, which mostly retains its chemical composition, while crystals are the result of a partial decomposition or de-mixing of the medium, resulting in the precipitation and crystallisation of one single degradation product or component. Thus, spectroscopic methods are ideal tools to distinguish these effects. Both the microscopic resolution and the molecular or structural information provided by Raman microspectroscopy make this technique particularly suited for such studies. In the case of cracks, a mostly unchanged Raman spectrum (representing the mixture of ingredients of the mounting medium) should be expected, while crystals should yield the spectroscopic signature of one degradation product or component of the medium.

**Figure 3:**
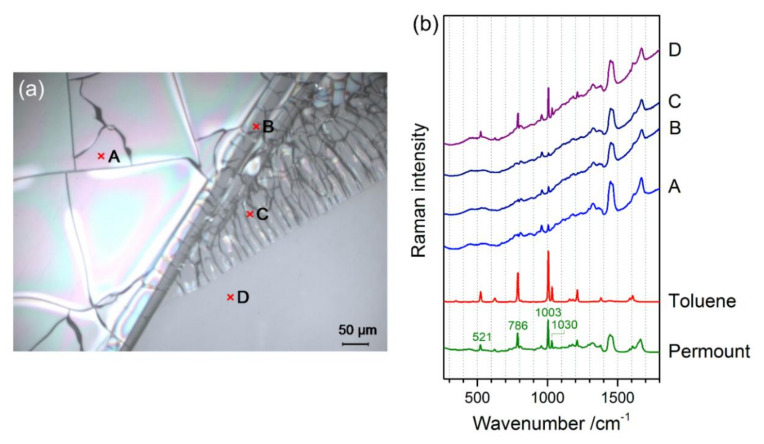
Determination of the reason for crack formation in Permount™ by Raman microscopy: (**a**) reflected light micrograph, and (**b**) Raman spectra of the Permount™ mounting medium, the solvent toluene, and of the measurement spots (**A**–**D**) in sample USNM 1226533, *Cateria gerlachi*, marked in (**a**).

**Figure 4:**
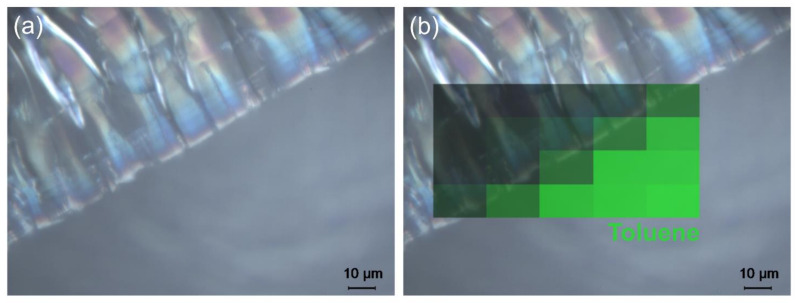
(**a**) Reflected light micrograph of sample USNM 1226533, *Cateria gerlachi*, without and (**b**) with a Raman distribution map of toluene overlaid (based on the intensity of the most prominent band).

As shown in Figure 3a, four spots (A–D) were selected on the sample USNM 1226533, *Cateria gerlachi* in Permount^TM^, from the collection of the United States National Museum, Washington, D.C., Smithsonian Institution, for Raman measurements, including spots on homogeneous (D) and crackly areas (A–C). For interpretation of the four Raman spectra in Figure 3b, the spectra of pure Permount^TM^ and its solvent toluene are shown for comparison. As previously shown by Schmid et al. [24], the Raman spectra of mounting media are superpositions of the spectral signatures of their components, and thus after the assignment of bands to a specific compound, this technique can be employed to monitor changes in chemical composition. Comparison of the toluene and Permount^TM^ spectra yields several sharp and strong bands of Permount^TM^, which are obviously due to toluene—for example, the peaks at 1030 cm^−1^, 1003 cm^−1^, 786 cm^−1^, and 521 cm^−1^ were due to vibrations of the aromatic ring. Except for the distorted baseline, which was due to autofluorescence emission, the spectrum D acquired on a homogeneous part of the sample resembles closely the spectrum of pure Permount^TM^, whereas on all crackly areas of the sample (A–C), loss of the solvent toluene can be observed. Only the most prominent band of toluene at 1003 cm^−1^ is visible in spectra A–C. Thus, we can conclude that crack formation is related to the (partial) evaporation of the solvent. This effect might include some feedback, in the sense that evaporation of the solvent changes the viscosity of the medium, leading to crack formation, and further evaporation of the solvent is facilitated by the enlarged surface areas at those cracks. The Raman map shown in Figure 4 confirms this observation, revealing that in the 5 × 4 spectra acquired on the surface of the sample USNM 1226533, *Cateria gerlachi* in Permount^TM^, only the ones from the homogeneous area contain a significant contribution from toluene.

**Figure 5:**
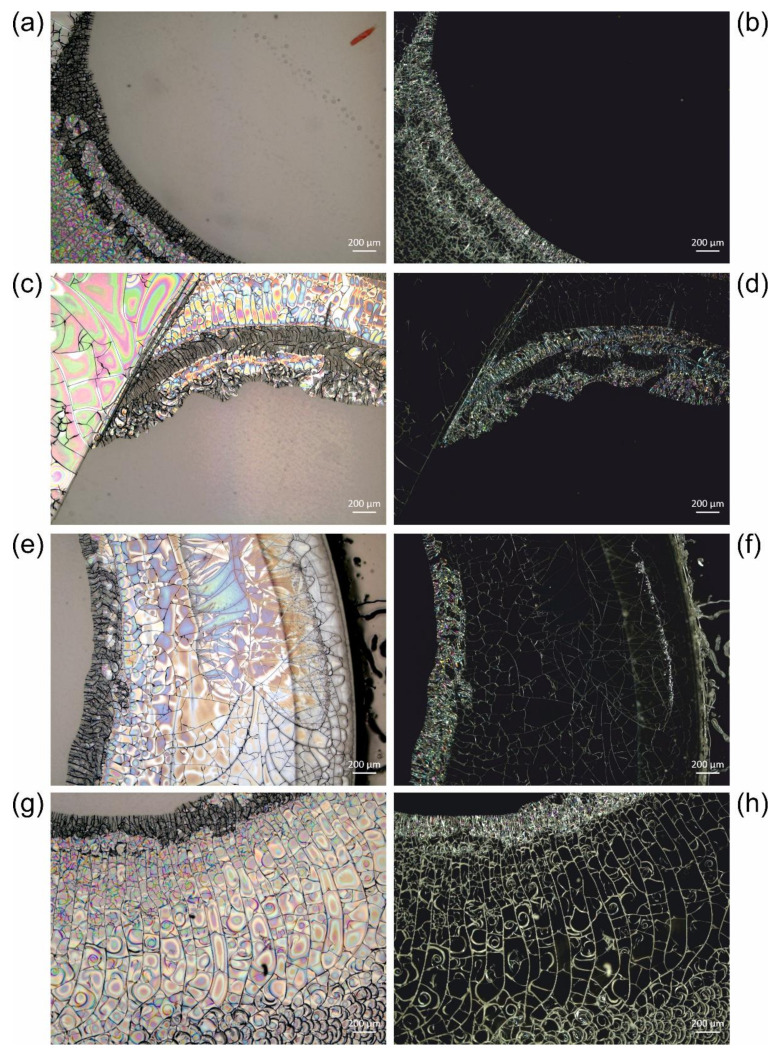
Different examples of crack formation in Permount™, sample USNM 1226533, *Cateria gerlachi*: (**a**,**c**,**e**,**g**) reflected light brightfield and (**b**,**d**,**f**,**h**) reflected light darkfield micrographs.

Figure 5 reveals different crackly parts of the sample USNM 1226533, *Cateria gerlachi* in Permount^TM^. The animal stained red seen in the top right corner of Figure 5a is surrounded by a ring of cracks, which reaches the edge of the cover slip (the right edge can be seen in Figure 5e,f). The cracks can be best seen in darkfield images, while the normal brightfield reflected light images additionally show various colours distributed over the slabs between the cracks. These are obviously effects of light diffraction and interference as a consequence of local detachment of the medium from the cover slip. The additional gas phase between the cover glass and the medium induces an additional change in the refractive index, leading to these optical effects. Most probably, the gas phase mainly consists of the locally evaporated toluene and/or air diffused into the medium from the edges.

**Figure 6:**
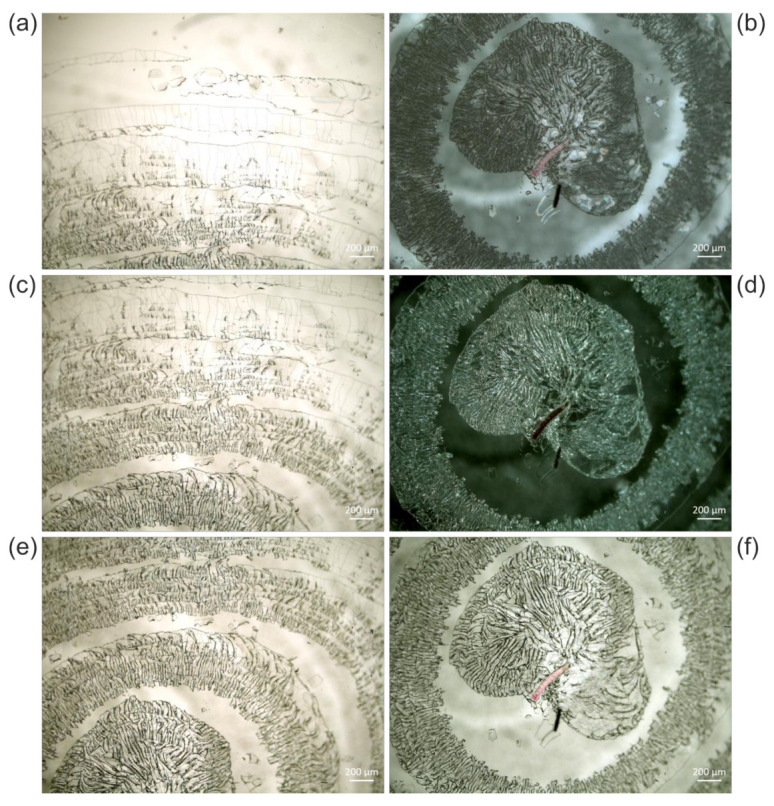
(**a**,**c**,**e**) Transmitted light micrographs of sample USNM 1226536, *Cateria gerlachi*, in Permount™, from the edge of the cover slip towards the centre showing cracks in concentric rings, and (**b**,**d**,**f**) the centre of the sample acquired in (**b**) reflected light brightfield, (**d**) reflected light darkfield illumination, and (**f**) transmitted light.

**Figure 7:**
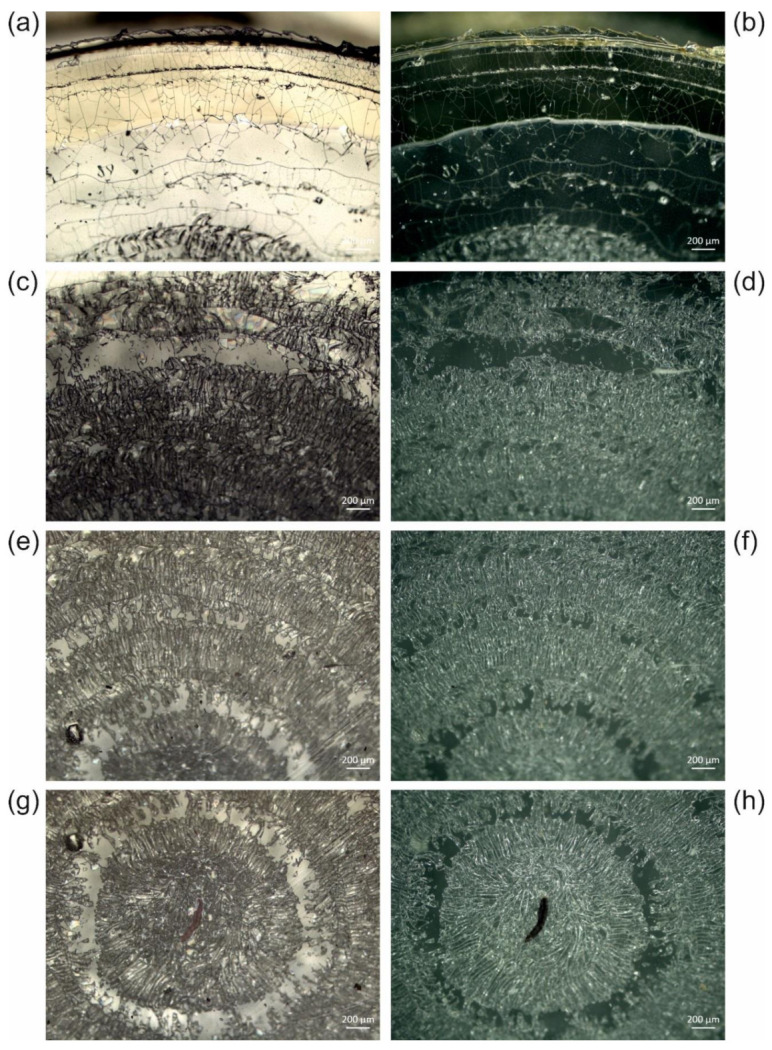
(**a**,**c**,**e**,**g**) Reflected light brightfield and (**b**,**d**,**f**,**h**) reflected light darkfield micrographs of sample USNM 1226550, *Cateria gerlachi*, in Permount™, from the edge of the cover slip (**a**,**b**) towards the centre (**g**,**h**), showing cracks in concentric rings with stronger interconnections than in sample USNM 1226536, *Cateria gerlachi* (Figure 6). Due to high optical density, transmitted light images were not accessible.

**Figure 8:**
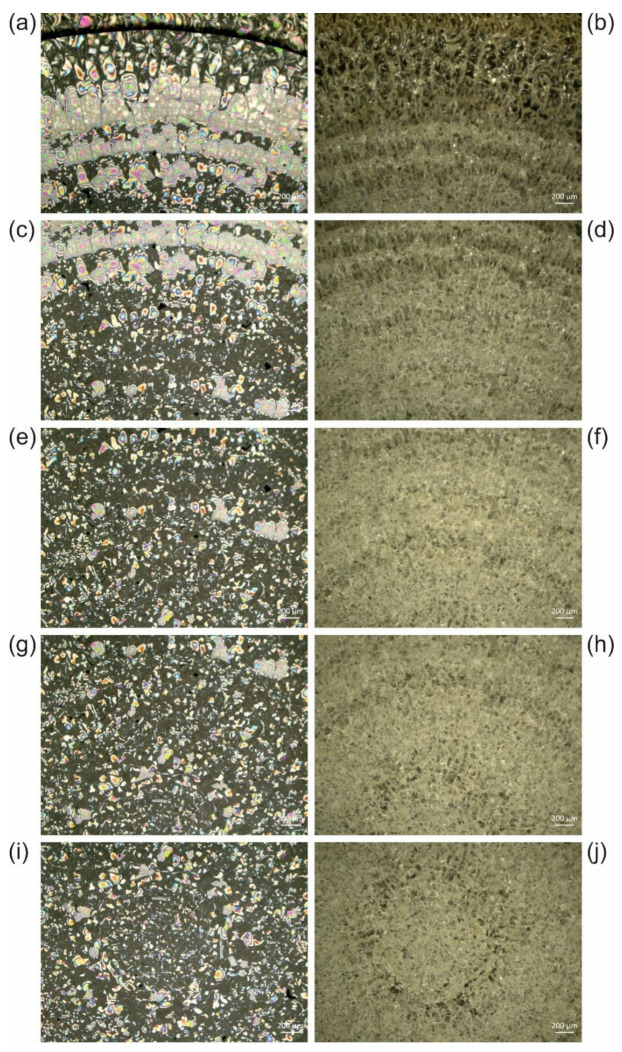
(**a**,**c**,**e**,**g**,**i**) Reflected light brightfield and (**b**,**d**,**f**,**h**,**j**) reflected light darkfield micrographs of sample USNM 1226524, *Cateria gerlachi*, in Permount™, from the edge of the cover slip (**a**,**b**) towards the centre (**i**,**j**), showing cracks in concentric rings almost completely connected to each other. Due to high optical density, transmitted light images were not accessible.

We observed different extents of crack formation. In the example revealed by Figure 5, only one ring with cracks reaching the edge of the sample has formed, without interfering with the animal. In the series of *Cateria gerlachi* in Permount^TM^ samples USNM 1226536 (Figure 6) < USNM 1226550 (Figure 7) < USNM 1226524 (Figure 8), we can follow three different stages of ageing in media containing concentric rings (with the animal in the centre within a circular crackly “island”) involving alternating crackly and flat areas. Such crackly structures forming concentric rings are also shown in Figure 17 in Reference [3]. Neuhaus and Kegel misinterpreted these cracks as crystals [18]. They also reported that cracks did not stop at the specimen but went right through them. Any restoration efforts lead to pieces of the specimen which are extremely difficult to study.

We suppose that this type of crack formation is triggered by mechanical stress applied to a sample. We would like to point out that all USNM samples discussed in this study were mounted between a circular and a rectangular coverslip placed in a metal frame for sample handling. Thus, mechanical force applied to one of the coverslips easily bends the whole sample and thus locally applies different shear forces to the medium–glass interface, leading to local detachment of the medium from the glass. As described by Neuhaus et al. [23], such mechanical stress can be due to observation of the specimen by using an oil-immersion objective (with direct contact between glass slide, immersion oil, and objective) and the necessary cleaning of the sample’s surface afterwards. Within two weeks after such events, the authors observed crack formation in previously crack-free samples, and the crack distribution was very similar to the example shown here in Figure 5.

In the samples shown in Figure 6, Figure 7 and Figure 8, concentric rings of alternating crackly and homogeneous areas have formed. In Figure 6, two types of cracks can be clearly distinguished, which are also present in other examples. Type 1 cracks are thin (or less deep than type 2, respectively) and form mosaic-like slabs. These can be seen in the centre of the sample (middle of the central crackly “island”, top right of the animal) and predominantly towards the outer part of the sample (Figure 6a). We suppose that type 1 cracks are formed first. The mechanical stress acting on them predetermines the direction of the formation of the thicker (or deeper, respectively) cracks of type 2, which are either radially arranged (Figure 6) or form rounded, shell-shaped slabs (Figure 5g,h). Decreases in the appearance of type 2 cracks in the radial direction towards the edge of the cover slip (Figure 6) clearly indicates their growing direction, away from the initial trigger in the centre. Here, the animal as a potential source of mechanical stress also comes into play. If the animal is thicker than the surrounding medium, it can induce additional shear forces if a mechanical trigger is applied to the sample. In fact, according to the radial arrangement of the cracks, the centre of their formation in Figure 6 seems to be directly next to the animal.

Once the distribution of cracks is predetermined by the applied force and the initial cracks of type 1, ageing of such altered glass slide samples leads to the formation of type 2 cracks (Figure 6), which can increasingly interconnect the initial crackly areas. This happens due to mechanical stress at the edges of the crackly areas, which in turn is caused by further evaporation of the solvent and further local detachment of the cover slip. Figure 7 and Figure 8 show this process in examples with initial concentric crackly rings, which eventually led to a sample completely covered with cracks, making observation of the animal impossible, which is also impaired by the partial detachment of the cover slip, leading to local changes of the refractive index.

### 3.3. Accelerated Ageing of Mounting Media

Accelerated ageing studies are based on the dependence of reaction kinetics on the temperature, as described by the Arrhenius equation [31]:

*k* = *C* exp(−*E*_a_/*RT*), (3)

with *k* denoting the reaction rate, *C* a pre-exponential factor, *E*_a_ the activation energy of the reaction, *R* the universal gas constant, and *T* the temperature. Rearrangement and substitutions convert the Arrhenius equation into the empirical 10-degree law, stating that an increase in temperature of 10 K doubles the reaction rate (approximately valid for reactions with reference to room temperature and activation energies <70 kJ/mol) [32,33]:

*k* = *C’* 2^((*T*−*T*^^ref^^)/10)^, (4)

with *C’* denoting a (different) pre-exponential factor, while *T* and *T*_ref_ are the actual and reference temperatures, respectively. For accelerated ageing studies investigating storage effects, *T*_ref_ is typically the room temperature, and the 10-degree law enables estimation of the acceleration of ageing effects at an artificially elevated temperature *T*. Within the present study, ageing experiments studying the effects of the atmosphere on the mounting media were conducted, according to Equation (4), at elevated temperatures of 40 °C, 60 °C, and 80 °C, accelerating ageing with respect to 20 °C by approximate factors of 4, 16, and 64, respectively.

#### 3.3.1. UV Irradiation

Ageing under the influence of light was accelerated by (1) employing ultraviolet (UV) radiation, and (2) using no coverslip, thus avoiding the partial absorption of UV radiation by glass. Samples of Canada balsam and Permount^TM^ were placed under an intense UV source and irradiated with a spectrum centred at 365 nm with a power density of 1350 mW/cm^2^ for 1 h. UV–Vis and Raman spectra of each sample were measured prior to and after irradiation. For comparison, yellowing and light scattering factors were calculated according to Equations (1) and (2).

**Figure 9:**
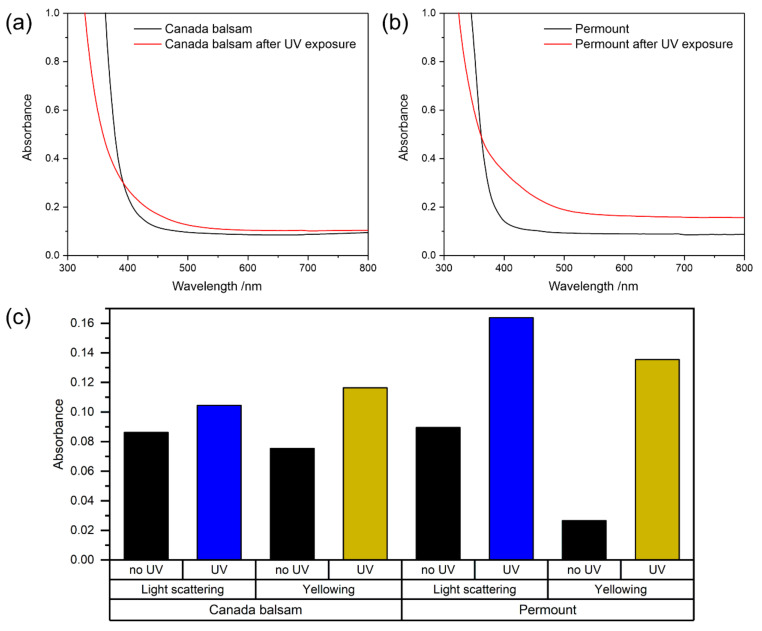
UV–Vis spectra of (**a**) Canada balsam and (**b**) Permount™ before and after exposure to UV radiation. In (**c**), measures for the extent of light scattering and yellowing derived from the spectra (**a**,**b**) are shown (see text for explanations).

As can be seen in Figure 9a,b, UV irradiation leads to a partial decrease of absorbance in both media in the UV range <400 nm. The effect is more pronounced in Canada balsam. Typical UV absorbers are organic molecules containing double bonds. The decrease in absorbance can be explained by the partial degradation of these molecules upon excitation by UV photons, i.e., by the bleaching of UV chromophores. At the same time, an increase in absorbance in the visible range can be observed for both media. Here, both media behave differently. While only a slight increase around 420 nm and no significant increase at higher wavelengths is seen in Canada balsam, the spectrum of Permount^TM^ shows both: an increase at around 420 nm together with a strong change of the slope of the spectrum in this range, and an increase at higher wavenumbers >550 nm.

An increase in absorbance at around 420 nm, i.e., in the violet spectral range, leads to the appearance of the complementary colour yellow. Also, the yellowing factor in Figure 9c shows that UV irradiation induced yellowing in both media. This only slightly increased in Canada balsam, which appeared to be yellow already at the beginning of the experiment, while a clearly visible and measurable change of colour appeared in the previously colourless Permount^TM^ medium. Additionally, a clear difference in terms of light scattering can be observed, which can be described quantitatively by measuring the absorbance at 600 nm. A particularly strong increase of light scattering can be seen in Permount^TM^, which is explained by the formation of cracks all over the surface visible to the naked eye. Such cracks and their microscopic observation are also discussed below in the section on accelerated oxidative ageing.

**Figure 10:**
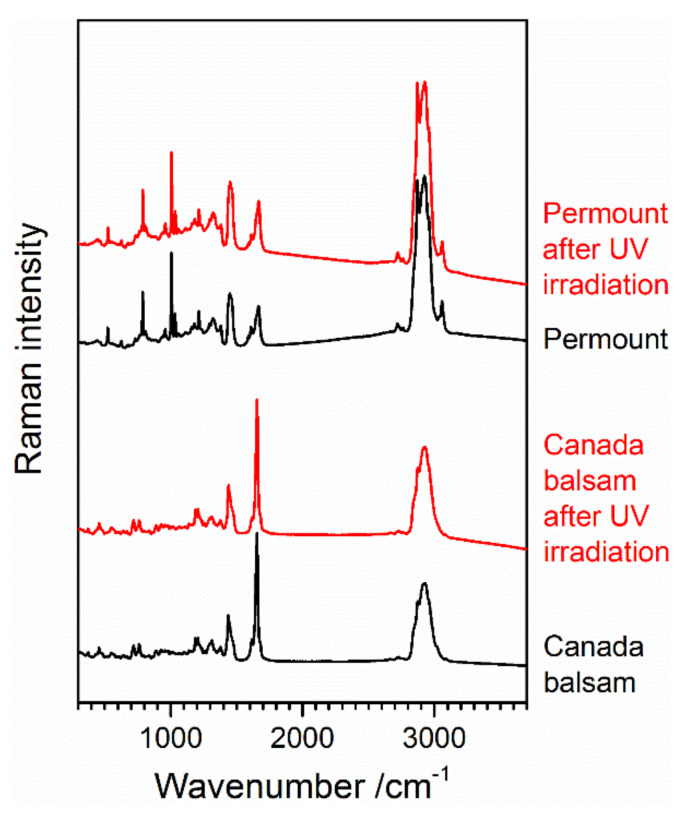
Raman spectra of Canada balsam and Permount™ before and after exposure to UV radiation.

Raman spectroscopy can give insight into chemical changes during accelerated ageing. In the present case, the spectra recorded prior to and after UV irradiation show no significant differences for both Canada balsam and Permount^TM^, as can be seen in Figure 10. As discussed in Section 3.2.2, the evaporation of the solvent toluene from Permount^TM^ would lead to a significant decrease in or even a depletion of the sharp bands in the Raman spectrum assigned to the aromatic ring vibrations, with the most prominent band of toluene at 1003 cm^−1^. Obviously, no evaporation of the solvent or significant change in chemical composition of the main components of the media occurred during 1 h UV irradiation. Most likely, only the top layers of the medium were influenced by UV radiation due to its limited penetration depth. For example, in Canada balsam, the absorbance at 365 nm was 0.9 at the beginning of the experiment. Thus, during transmission from the air–medium interface down to the glass slide, the applied UV irradiation was attenuated to 10^−0.9^ = 13% of the initial value, and only as the experiment progressed, the penetration depth increased due to the described bleaching effect. We conclude that the observed yellowing effect was caused by only trace amounts of molecules chemically modified by the UV irradiation, and the major part of the medium remained chemically unchanged, but exposure to light might start chemical processes, which proceed even when storing samples in a dark environment afterwards [3,29] (p. 79).

#### 3.3.2. Oxidative Ageing at Elevated Temperatures

**Figure 11:**
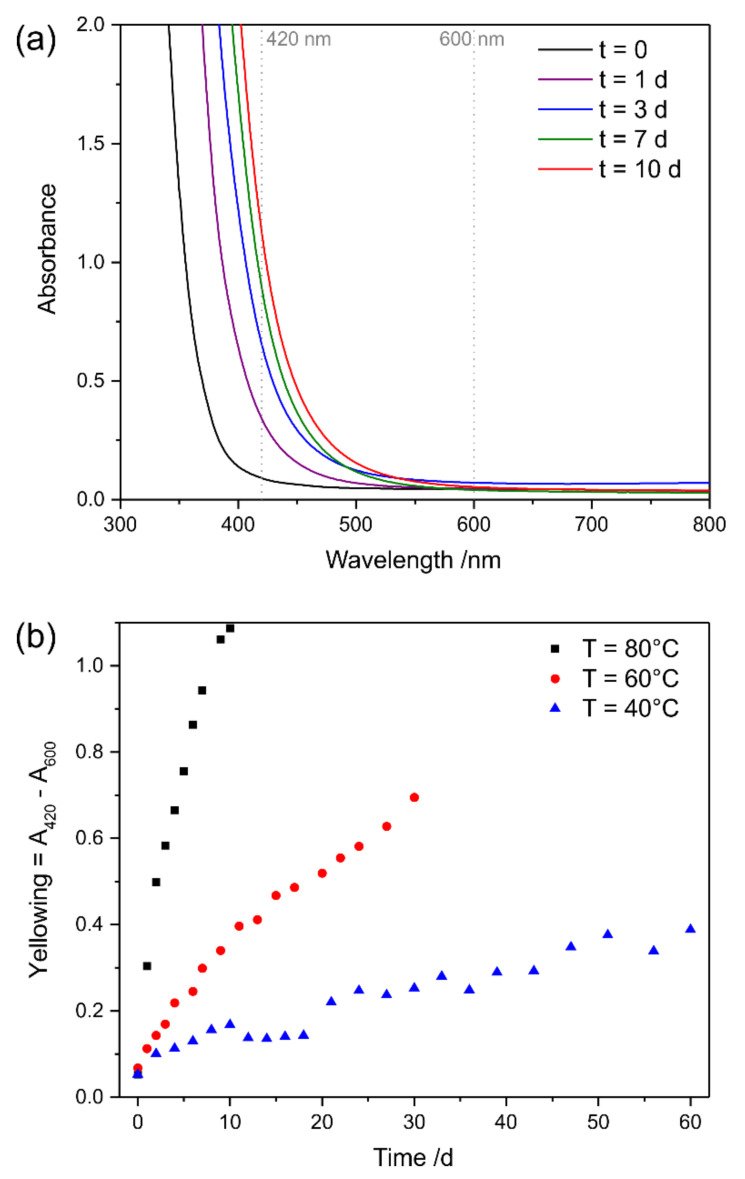
(**a**) UV–Vis spectra of Canada balsam after exposure to 80 °C for different numbers of days, and (**b**) development of the yellowing factors over time during exposure of Canada balsam to different temperatures.

The UV–Vis spectra of Canada balsam exposed to the atmosphere at 80 °C for 10 days show a shift of the edge of their optical absorption (with their not detectable maximum in the UV range) towards and into the visible light range during the progression of the experiment (see Figure 11), while the slope of the spectrum did not change. This leads to absorbance of the medium in the violet and, later, the blue spectral range, leading to a colour change to yellow, followed by orange. Raising of the baseline of the spectrum, which is an indicator of increasing light scattering, was not observed. For clarity, the wavelengths used for calculating yellowing and light scattering factors are marked in Figure 11 with dashed lines.

According to Equation (1), yellowing factors were calculated for the accelerated ageing experiments conducted at 40 °C, 60 °C and 80 °C, and are plotted in Figure 11b. An increase in yellow colour was observed at all temperatures; only the reaction rates differed. When assuming simple first-order kinetics, reaction rates can be derived from the determined yellowing factors, as described in Appendix A. An increase by a factor of 2.8 is observed for the temperature increase from 40 °C to 60 °C, and a factor of 4.4 describes the step from 60 °C to 80 °C. Thus, the data approximately fulfils the 10-degree law according to Equation (4), which predicted a factor of 4 for both steps. Assuming the 10-degree law, the acceleration factors, with regard to a reference temperature of 20 °C, are 4, 16, and 64, respectively, for the three temperatures under investigation. The values determined at the end of the experiments, after 60 days (40 °C), 30 days (60 °C), and 10 days (80 °C), respectively, therefore relate to the yellowing factors expected to be observed after 240 days (8 months), 480 days (approx. 1 year 4 months), and 640 days (approx. 1 year 9 months), respectively, when stored at room temperature. We would like to point out that, as in the UV experiments described above, further acceleration with respect to a typical glass slide sample was achieved by preparing the mounting media without cover slips. With the reaction rates and their known temperature dependency determined according to Equation (3), the activation energy of this yellowing reaction can be determined, and the Arrhenius plot yields a value of 58 kJ/mol (see Appendix A).

**Figure 12:**
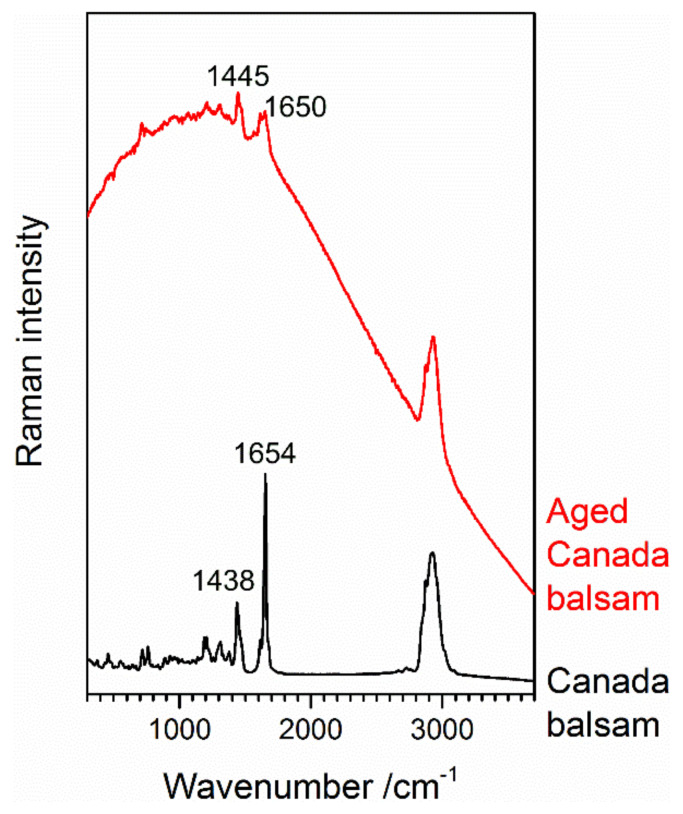
Raman spectra of Canada balsam before and after accelerated ageing at T = 80 °C for 10 days.

The yellowing factor of Canada balsam during accelerated oxidative ageing reached the level observed after UV exposure (see above) after 2 days at 40 °C and 1 day at 60 °C, respectively. It was 1 order of magnitude higher after 10 days at 80 °C. Thus, also more pronounced changes in chemical composition were expected. Figure 12 shows the Raman spectra of Canada balsam at the beginning and at the end of the 10-day ageing experiment conducted at 80 °C. There are two main changes observed in the spectra. First, a strongly increased and shaped baseline is observed, due to fluorescence emission by the medium. The maximum of the baseline at approx. 1140 cm^−1^ is the centre of a fluorescence band at an emission wavelength of 682 nm in the red spectral range. This is a typical effect of coloured samples. As they absorb visible light, they can also be prone to fluorescence emission, which can be excited in this spectral range and can interfere with the Raman spectra acquired in the same range. Obviously, an increase in yellowing causes a rise in fluorescence emission.

**Figure 13:**
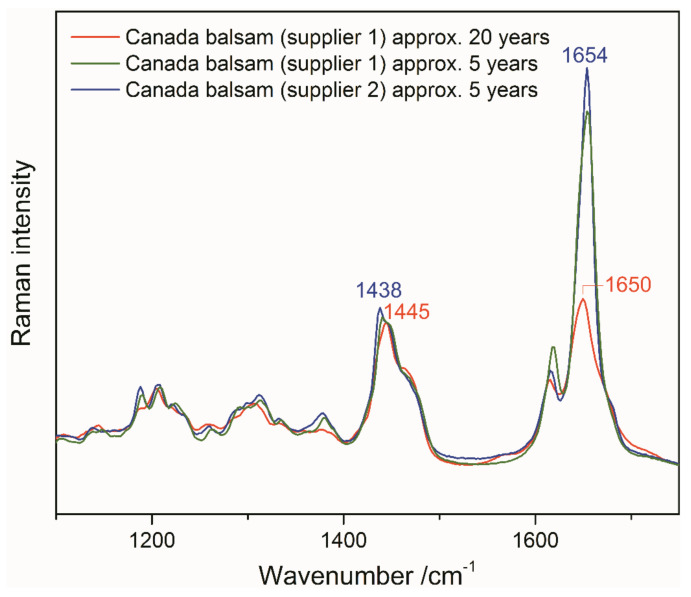
Raman spectra of Canada balsam samples from two different suppliers after different shelf storage times.

The second obvious spectral change is a strong decrease of the sharp and most prominent Raman band of Canada balsam at 1654 cm^−1^, accompanied by shifts of this and the second strongest band at 1438 cm^−1^. This brings us to a close look at three batches of Canada balsam stored in the laboratory of the Museum für Naturkunde Berlin for different periods of time. Figure 13 shows baseline-corrected and scaled Raman spectra of Canada balsam from two suppliers, stored for approximately 5 and 20 years, respectively. The spectra of the 5-year samples resemble the black trace in Figure 12, with a pronounced and sharp band observed at 1654 cm^−1^ and the most prominent peak within several superimposed C–H bending modes seen at 1438 cm^−1^. Similar to the artificially aged Canada balsam (see the red trace in Figure 12), the 20-year batch reveals the most intense C–H bending Raman band at 1445 cm^−1^ and a C=C stretch vibrational mode at 1650 cm^−1^ with significantly reduced intensity.

As mentioned above at the end of Section 3.2.1, the observed yellowing of aged Canada balsam, in accordance with studies on fossil resins, might be related to the aromatisation of aliphatic or olefinic terpenoid ring systems. Further to this process, polymerisation leads to a loss of olefinic (non-aromatic) double bonds. The resulting reduction of the intensity ratio of the olefinic C=C stretch vibrational mode in Raman spectra at approx. 1640 cm^−1^ and a C–H bending mode of aliphatic groups at approx. 1440 cm^−1^ was proposed as an age indicator of fossil (ambers) and subfossil resins (copals) [27]. In a study on Raman spectroscopy of fossil resins, Naglik et al. critically discussed this ageing index, because polymerisation and aromatisation depend on several environmental factors (e.g., temperature and irradiance) beyond just the resin’s age, and therefore termed the ν(C=C)/δ(C–H) ratio more precisely as an indicator of maturation grade [28]. Accordingly, the maturation of Canada balsam samples in the present study is indicated not only by yellowing (quantifiable as the yellowing factor extracted from UV–Vis spectra) but also by the significant decrease in the relative intensity of the sharp Raman band at 1654 cm^−1^ assigned to olefinic ν(C=C) vibrations. Shifts of ν(C=C) and δ(C–H) vibrational frequencies furthermore indicate chemical transformations. The same variation in the relative intensity of the ν(C=C) Raman band can be also observed in the spectra of the historical Insecta and Crustracea samples shown in Figure 1. Samples *Stylocheiron longicorne* and Crustacea reveal the weakest ν(C=C) bands along with the highest yellowing degrees.

**Figure 14:**
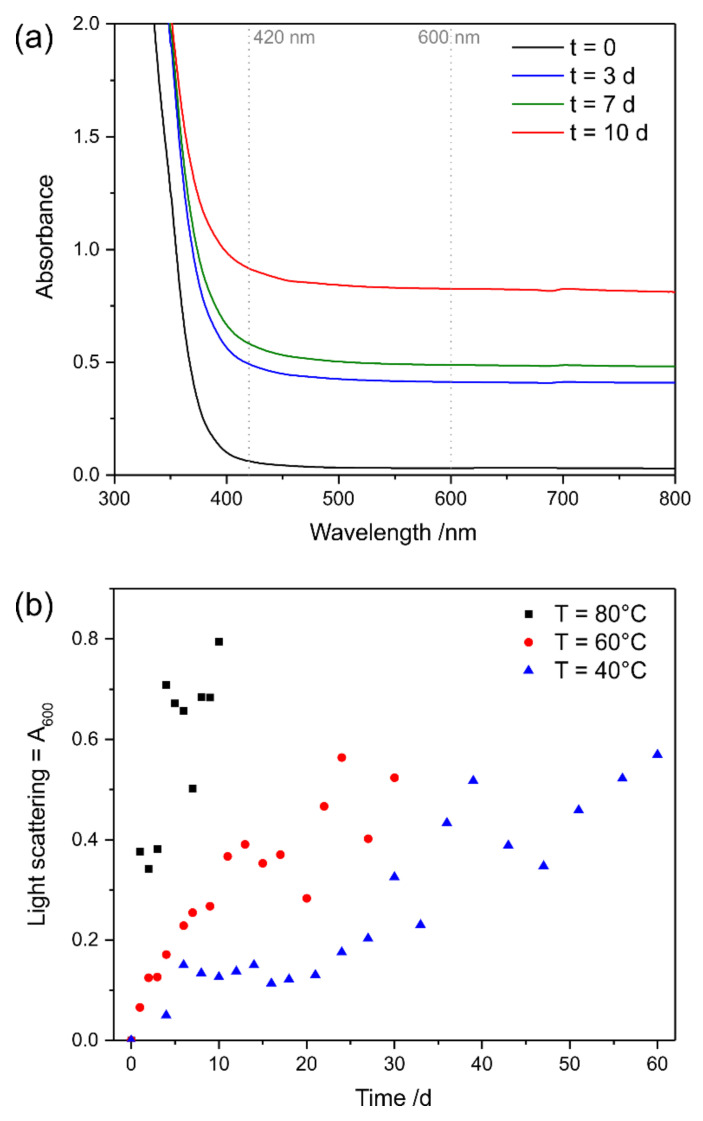
(**a**) UV–Vis spectra of Permount™ after exposure to 80 °C for different numbers of days, and (**b**) development of the light scattering factors over time during exposure of Permount™ to different temperatures.

**Figure 15:**
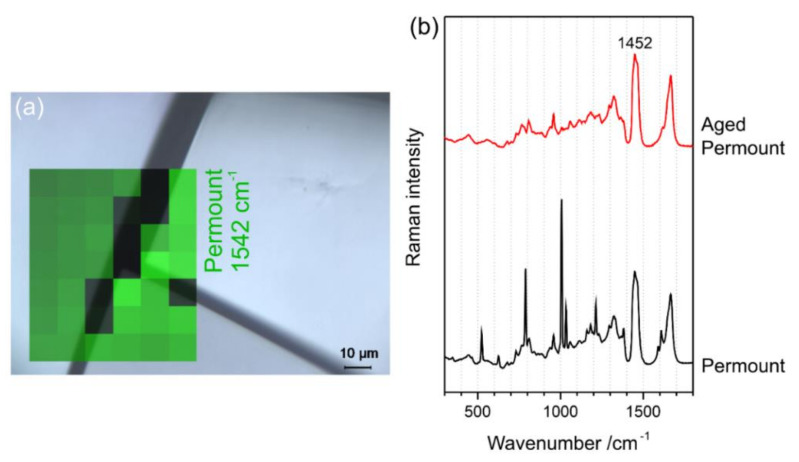
(**a**) Reflected light micrograph of Permount™ after accelerated ageing at T = 80 °C for 10 days overlaid with the Raman distribution map of Permount™, and (**b**) Raman spectra of this aged and of pristine Permount™.

Accelerated ageing experiments under oxidative conditions with Permount^TM^ yield a very different picture. While the shapes of the UV–Vis spectra acquired throughout the experiment remain practically unchanged, the spectra appear shifted parallel to the abscissa by offsets, which increase over time (see Figure 14a). The results of this behaviour are constant yellowing factors, i.e., according to Equation (1), unchanged differences between the absorbances at 420 nm and 600 nm, and steadily increasing light scattering factors, which corresponding to the definition in Equation (2), are expressed by the increasing absorbance at 600 nm (see Figure 14b). Figure 15 reveals cracks in the mounting medium as the reason for light scattering. While Raman mapping can easily confirm the straight structures as empty cracks (Figure 15a), the Raman spectrum of the aged Permount^TM^ medium significantly differs from the fresh medium, because of the loss of the sharp Raman bands related to the solvent toluene (Figure 15b). These observations are in accordance with the investigation of the historical samples mounted in Permount^TM^ discussed above in Section 3.2.2.

## 4. Conclusions

The present study revealed very different ageing behaviours of Canada balsam and Permount^TM^. While Canada balsam was subject to purely chemical conversion reactions leading to increasing colouration, Permount^TM^ underwent physical property changes such as crack formation, which caused light scattering and diffraction phenomena. The latter were shown to be much more deleterious to microscope slide samples within a relatively short period of time, because they were able to even crack the specimen and make microscopic observation almost or totally impossible. However, in the long run, the chemical changes to Canada balsam may lead to polymerisation of the medium, making dissolution of the medium by organic solvents in the process of restoration efforts unfeasible.

Intriguingly, the investigations of both historical microscope slide samples and artificially aged mounting media on test slides yielded consistent results: the purely chemical ageing of Canada balsam and the physical degradation of Permount^TM^, which underlined the appropriateness of the approach. Furthermore, the combined application of microscopic observation with spectroscopic measurements using a UV–Vis spectrometer and a Raman microscope turned out to be successful for studying the effects either complicating or hampering the microscopic observation of the animal, and for gaining insights into their physical and chemical reasons.

Canada balsam has, despite its yellowing over time, been suggested to belong to the long-lasting, non-deteriorating, and most recommendable mounting media category for microscope slides, whereas Permount^TM^ has long been known to deteriorate considerably within years (see reviews by Brown [1] and Neuhaus et al. [3], as well as Refs. [7,8]). The colour change in Canada balsam has been euphemistically termed yellowing, but after sufficient time, the entire medium will darken to such a degree that observation with transmitted light becomes quite challenging (Figure 2d; pers. obs.). The observed chemical changes of Canada balsam, both by natural and by our experimental ageing, raise doubts over whether this medium should still be regarded as lasting for centuries. Of course, in comparison with media like Permount^TM^, which degrades to unusable slides within a few years, Canada balsam still represents one of the longest lasting media.

## 5. Outlook

**Figure 16:**
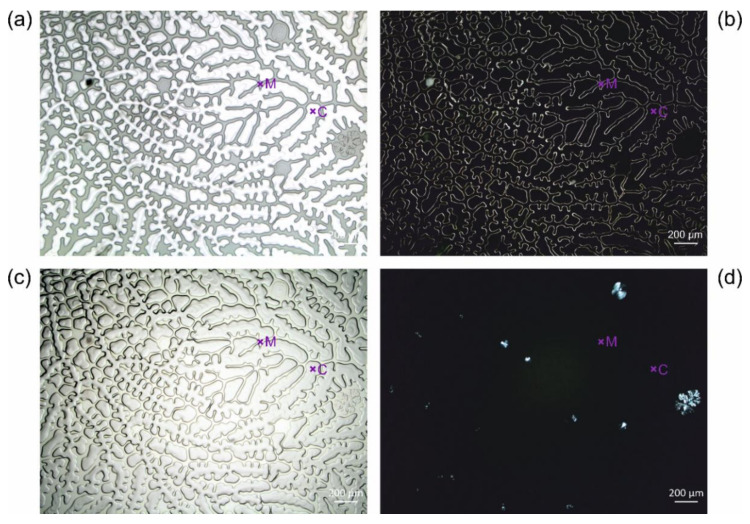
Light micrographs of cavities in an unknown mounting medium, sample USNM 1226589, *Cateria styx*: (**a**) reflected light brightfield, (**b**) reflected light darkfield, (**c**) transmitted light micrograph with parallel, and (**d**) crossed polarisers. According to the assignments derived from the micropectroscopic maps shown in Figure 18e–g, ‘M’ and ‘C’ exemplarily mark medium and caverns, respectively.

Our investigation of another microscope slide sample from the collection of United States National Museum, Washington, D.C., Smithsonian Institution, reveals further possible damages as well as limitations, and the further potential of Raman spectroscopy within this field of research. The sample USNM 1226589, *Cateria styx*, was found to be pervaded by rounded caverns and to contain some crystal-like structures (see Figure 16). Unfortunately, the mounting medium could not be identified by Raman spectroscopy, because of hampering autofluorescence emission. As Raman bands always appear at the same wavenumbers shifted from the laser frequency currently employed for excitation, while emission by a certain fluorophore is linked to a specific wavelength, Raman spectra can be shifted to another wavelength range in the case of disturbing fluorescence by changing the laser excitation source. In the present case, the whole spectral range accessible by the available laser sources (532 nm, 632.8 nm and 785 nm) was strongly affected by the fluorescence emitted by the aged mounting medium, making the acquisition of its Raman spectrum impossible. This raises the need for alternative spectroscopic techniques which can be applied in cases of strongly fluorescing microscope slides. Infrared (FTIR or ATR-FTIR) spectroscopy is complementary to Raman spectroscopy in the sense that while it also delivers vibrational spectra and, therefore, similarly specific molecular information, it is not influenced by visible fluorescence emission (because it is performed in the infrared range) and yields bands with different relative intensities (because of different selection rules). Due to the latter, O–H vibrational bands—in contrast with Raman spectra—are very strong in FTIR spectra, in most cases making measurements in aqueous media and through glass slides impossible. Thus, spectroscopies in the infrared range can be used forthe identification of strongly fluorescing media, but (a part of) the coverslip may have to be removed for analysis if no portion of the medium has escaped outside the coverslip, making this a (partly) destructive approach. The method has been applied successfully by di Giacomo, but comparative spectra of mounting media have not been published yet [22].

**Figure 17:**
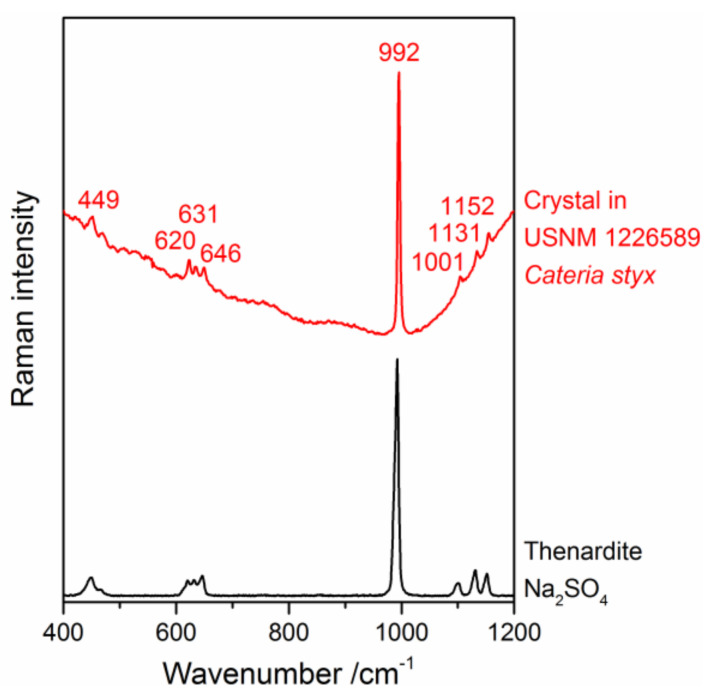
Raman spectrum of a crystal found in sample USNM 1226589, *Cateria styx*, in an unknown mounting medium (red) in comparison with the reference spectrum of the mineral thenardite (Na_2_SO_4_, black) from the RRUFF spectral database library [34].

**Figure 18:**
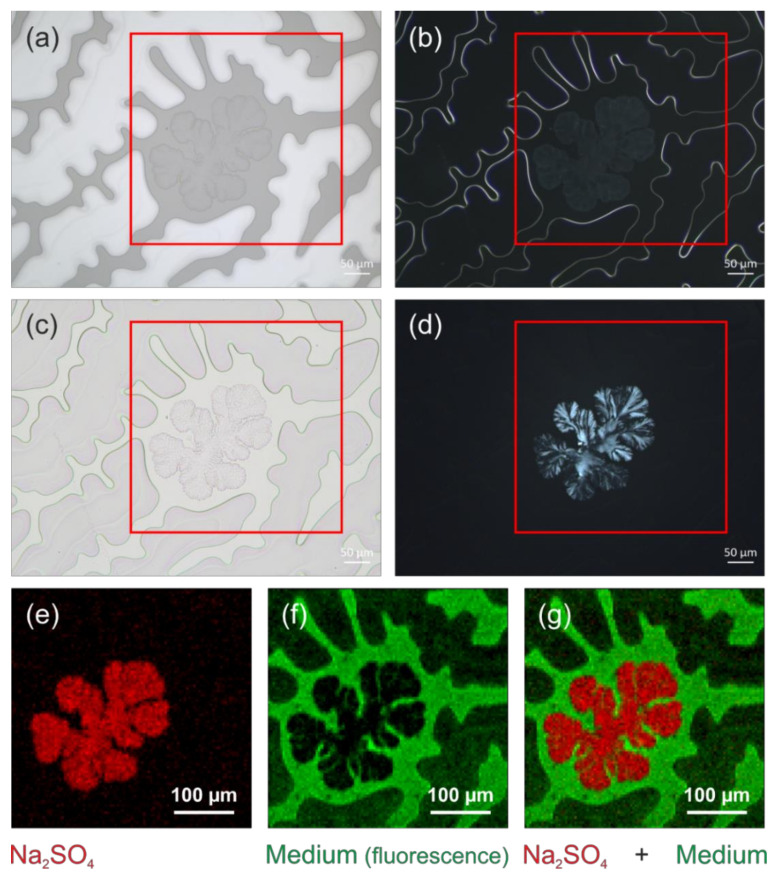
Example of crystal precipitation in an unknown mounting medium, sample USNM 1226589, *Cateria styx*: (**a**) reflected light brightfield, (**b**) reflected light darkfield, and (**c**) transmitted light micrograph with parallel and (**d**) crossed polarisers, (**e**) Raman distribution map of Na_2_SO_4_ (based on the intensity of the most prominent Raman band of the mineral thenardite), (**f**) distribution map of medium (based on the intensity distribution of its autofluorescence emission), and (**g**) overlay of both distribution maps.

As further research is needed to identify this mounting medium, we focused on investigating the observed damages, which differ from yellowing and the crack formation described above, and thus extend the catalogue of damages. While different imaging contrasts in light microscopy reveal different aspects of the structures within the mounting medium, only a microspectroscopic measurement can prove which structures are still filled with the medium, which represent caverns, and what the composition of the crystal-like structures is. The chemical nature of the latter was determined as thenardite or Na_2_SO_4_, respectively, by a “fingerprint” comparison of their Raman spectra with reference data taken from a spectral database library [34], as shown in Figure 17. Because the medium cannot be identified by Raman spectroscopy, its distribution was mapped based on the intensity of its unspecific autofluorescence, while thenardite was localised by plotting the intensity of its most prominent Raman band appearing at 992 cm^−1^ (see Figure 18). Both spectral intensities were acquired with the same Raman microscope and during the same experiment. The reason for the addition of this sodium sulphate to the mounting medium (perhaps dissolved in water prior to precipitation) remains unclear, but the structures can now be understood as medium with caverns filled either with air or (in a few spots) with Na_2_SO_4_ crystals.

## Figures and Tables

**Table 1:** Origin of specimens studied.

Supraspecific Taxon	Species	Catalogue Number	Deposition Site	Location
Kinorhyncha	*Cateria gerlachi* Higgins, 1968 [25]	1226524	United States National Museum, Washington, D.C., Smithsonian Institution	North Indian Ocean, Bay of Bengal, India, Waltair, beach near Palm Beach Rocks
		1226531		
		1226533		
		1226536		
		1226547		
		1226549		North Indian Ocean, Bay of Bengal, India, beach near Waltair, 17°50′ N, 083°15′ E
Insecta	*Ceroplastes formicarius* Hempel, 1900	n. a.	Museum für Naturkunde Berlin	Brazil, Yparanga (=Ypiranga); mounted by T.K. Qin 1990
	*Forda formicaria* von Heyden, 1837	n. a.		Germany, Saxony, Sächsische Schweiz, from grass roots; mounted by Heymons about or after 1907
Crustacea	*Stylocheiron longicorne* Sars, 1883 (= *S. mastigophorum* G.O. Sars, 1883)	n. a.		Atlantic Ocean, 30°34.9′ S, 006°10.2′ E, 5108 m depth; mounted after 1899
	undetermined species	n. a.		n. a.
Mammalia	*Sorex Araneus* Linnaeus, 1758	142b	Hubrecht Collection from Naturalis Biodiversity Center, Leiden, temporarily given on loan to Museum für Naturkunde Berlin	n. a.
	*Ctenodactylus gundi* (Rothmann, 1776)	208a		Utrecht; mounted by A. Weiss 1971

**Figure A1:**
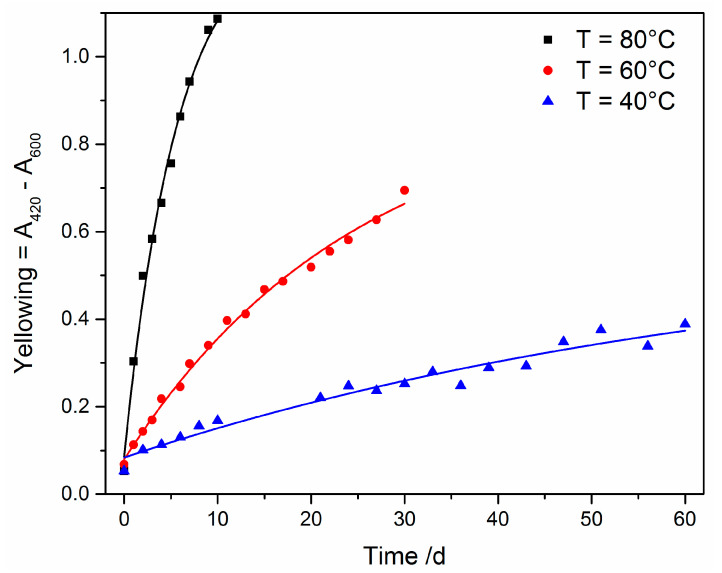
Data from accelerated oxidative ageing experiments with Canada balsam (as shown in Figure 11) with fitted trend lines according to Equation (A1).

**Figure A2:**
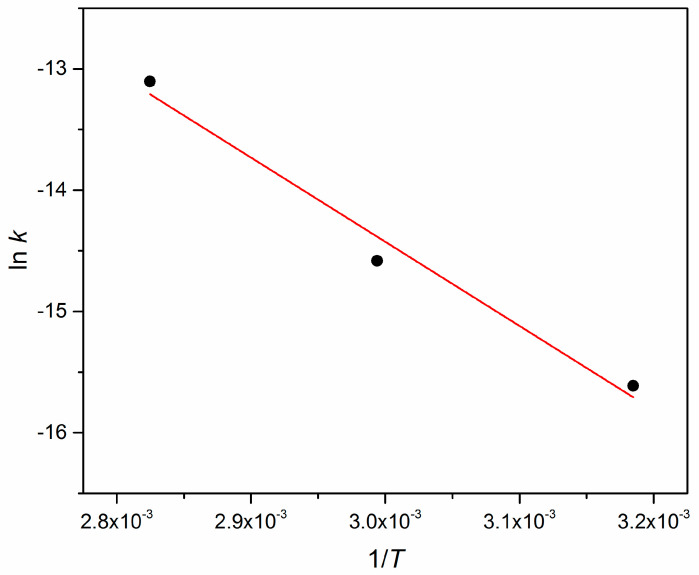
Arrhenius plot according to Equation (A2) of the accelerated oxidative ageing data of Canada balsam (R^2^ = 0.9812).

## Data Availability

The datasets generated during the current study are available from the corresponding author on reasonable request.

## Appendix A. Arrhenius Plot

Assuming simple first-order kinetics for the oxidative yellowing of Canada balsam, the temporal change of the yellowing factor *Y_t_* is expected to follow this equation:

*Y_t_* = *Y**_∞_* [1 − exp(−*k*(*t − t*_0_))], (A1)

with *k* denoting the reaction rate, *t* the time, *t*_0_ the starting time of the reaction, and *Y*_∞_ the (theoretical) end yellowing factor, which the sample asymptotically approaches. Fitting of Equation (A1) to the data shown in Figure 11 yields the trends revealed in Figure A1 and the reaction rates presented in Table A1.

Rearrangement of the Arrhenius equation in Equation (3) yields:

ln *k* = ln *A* − (*E*_a_/*R*)(1/*T*). (A2)

Thus, a plot of ln *k* as a function of (1/*T*) is expected to yield a linear trend with a negative slope of −(*E_a_*/*R*), enabling the determination of the activation energy. Figure A2 reveals such an Arrhenius plot for the three investigated temperatures.

**Table A1:** Reaction rates *k* and correlation coefficients *R*^2^ from the fitting of Equation (A1) to the oxidative ageing data of Canada balsam, as shown in Figure A1.

*T* [°C]	*k* [s^−1^]	*R* ^2^
40	1242	0.9571
60	3463	0.9938
80	15211	0.9907

The data in Table A1 show that the 10-degree law as explained by Equation (4) is approximately fulfilled, as the rates increase by an approximate factor of 4 for every 20-degree step. Also, the Arrhenius plot displays approximately linear behaviour. The activation energy of the yellowing of Canada balsam under the influence of the atmosphere derived from the slope of the Arrhenius fit is 58 kJ/mol, i.e., below 70 kJ/mol, underlining the validity of the 10-degree law [32,33].
